# Report-Documented Frequency and Spectrum of Incidental Findings on Emergency Abdominopelvic Computed Tomography: A Retrospective Clinical Data Study from Saudi Arabia

**DOI:** 10.3390/diagnostics16162649

**Published:** 2026-08-20

**Authors:** Turki M. Dhayihi, Yazeed B. Abutaleb, Mohammad A. Jareebi, Alanoud E. Dalak, Mousa Y. Wafi, Mada M. Jarad, Dekra Y. Arishi, Abdulaziz H. Alnadhari, Ebtisam A. Madkhali, Nadim I. Areshy, Mohammed K. Zughlul, Abdulwahab A. Aqeeli, Farjah H. Algahtani, Ghazi I. Al Jowf, Jalal Abu Halimah

**Affiliations:** 1 Department of Surgery, College of Medicine, Jazan University, Jazan 45142, Saudi Arabia; tdhayihi@jazanu.edu.sa (T.M.D.); jabuhalimah@jazanu.edu.sa (J.A.H.); 2 Faculty of Medicine, Jazan University, Jazan 45142, Saudi Arabia; moonar07@gmail.com (N.I.A.); zglool11@gmail.com (M.K.Z.); 3 Department of Family and Community Medicine, College of Medicine, Jazan University, Jazan 45142, Saudi Arabia; aaqeeli@jazanu.edu.sa; 4 Radiology Department, King Fahd Central Hospital, Jazan 45911, Saudi Arabia; alanood1620@gmail.com (A.E.D.); mousawafi@outlook.sa (M.Y.W.); dr.dekra.arishi@gmail.com (D.Y.A.); 1ebtisamahmed@gmail.com (E.A.M.); 5 Department of General Surgery, King Fahd Central Hospital, Jazan 45911, Saudi Arabia; mada1414@hotmail.com; 6 Radiology Department, Armed Forces Hospital Southern Region, Khamis Mushait 62413, Saudi Arabia; dr.abdulaziz.alnadhari@outlook.com; 7 Oncology Center, Chair of Epidemiology and Public Health Research, Faculty of Medicine, King Saud University/King Saud Medical City, Riyadh 12373, Saudi Arabia; falgahtani@ksu.edu.sa; 8 Department of Public Health, College of Applied Medical Sciences, University Medical Clinics Complex, King Faisal University, Al Hofuf 37912, Saudi Arabia

**Keywords:** incidental findings, computed tomography, emergency department, abdominopelvic imaging, Saudi Arabia, radiology reporting

## Abstract

**Background/Objectives**: Incidental findings (IFs) on emergency computed tomography (CT) are increasingly common as cross-sectional imaging utilization expands. Data from the Middle East are limited. We aimed to determine the report-documented frequency, anatomical distribution, and proportion of clinically actionable IFs on emergency abdominopelvic CT examinations at a tertiary-care center in southwestern Saudi Arabia. **Methods**: This retrospective cross-sectional study reviewed 705 consecutive emergency abdominopelvic CT examinations performed at King Fahd Central Hospital, Jazan, between 1 January 2023 and 31 December 2025. Two senior radiology residents (R3 and R4) extracted findings unrelated to the presenting indication from consultant-approved reports, applying a predefined terminology-standardization framework. Clinical actionability was defined a priori as a documented recommendation for further evaluation or follow-up. Descriptive statistics with 95% Wilson confidence intervals (CI) were calculated using R Software. **Results**: At least one IF was identified in 138 of 705 examinations, yielding a patient-level report-documented rate of 19.6% (95% CI 16.8% to 22.7%). A total of 255 IFs were recorded, averaging 1.85 findings per affected patient. The mean age was 42.3 ± 18.2 years; 78 patients (56.5%) were male and 116 (84.1%) were Saudi nationals. Findings were most often within the abdominopelvic cavity (159; 62.4%), followed by miscellaneous (64; 25.1%) and thoracic-vascular (32; 12.5%). At the secondary-category level, the most prevalent groups were spine (38; 14.9%), hepatobiliary (37; 14.5%), and genitourinary (31; 12.2%). Documented further work-up was recommended in 48 of 138 patients with IFs (34.8%; 95% CI 27.3% to 43.0%). **Conclusions**: Approximately one in five emergency abdominopelvic CT examinations contained incidental findings, with one-third requiring further evaluation. Spine, hepatobiliary, and genitourinary findings were most common. Variability in reported rates likely reflects differences in study methods and definitions. Future multicenter studies should evaluate standardized reporting approaches and follow-up strategies, together with their impact on reporting consistency, downstream investigations, and patient outcomes.

## 1. Introduction

Computed tomography (CT) has become a foundational tool in the rapid evaluation of patients presenting to emergency departments (EDs) [1]. In the United States, more than 62 million CT examinations are performed annually, and emergency utilization has increased by over 330% in recent decades [2]. Advances in scanner resolution and multidetector technology have shortened time to diagnosis but have also raised the rate at which clinicians encounter findings unrelated to the presenting complaint, generally referred to as incidental findings (IFs) [3]. Although many IFs are benign, a subset represents clinically meaningful pathology that may warrant additional investigation; their discovery can also generate patient anxiety, additional testing, and incremental healthcare costs [3,4].

The literature on IFs is dominated by studies from North America and Europe, often confined to specific clinical scenarios such as suspected renal colic or hematuria evaluation [4,5,6,7]. Reports from the Middle East are scarce, and few studies have systematically catalogued IFs across abdominal, genitourinary, gynecological, musculoskeletal, vascular, and thoracic systems within a single ED cohort [2]. Documentation in routine reports is also incomplete: in one urban ED, fewer than 10% of IFs were properly recorded in discharge paperwork [8], which highlights the need for better recognition, standardization, and reporting.

Although incidental findings on emergency CT examinations have been extensively investigated in North American, European, and other international populations [9], evidence from Saudi Arabia and the wider Middle Eastern region remains limited. Differences in patient demographics, disease patterns, healthcare systems, and radiology reporting practices may influence the frequency and reporting of incidental findings, limiting the generalizability of findings from other settings [10]. This study therefore aimed to characterize the report-documented frequency, anatomical distribution, and reported clinical actionability of incidental findings on emergency abdominopelvic CT examinations in a tertiary care hospital in Saudi Arabia, thereby providing region-specific data to inform future research and quality-improvement initiatives. By providing structured data from an under-represented region, this study seeks to inform local practice and to contribute to the global evidence base on the epidemiology and management of incidental imaging findings.

## 2. Materials and Methods

### 2.1. Study Design and Setting

This was a single-center, retrospective, cross-sectional study conducted in the Emergency Department of King Fahd Central Hospital, a tertiary-care referral center serving the Jazan region of southwestern Saudi Arabia. The study was reported in accordance with the Strengthening the Reporting of Observational Studies in Epidemiology (STROBE) statement [11]. CT examinations were performed on a 64-slice multidetector CT scanner (Siemens SOMATOM Definition, Siemens Healthineers, Erlangen, Germany). Standard emergency abdominopelvic protocols included both non-contrast and contrast-enhanced acquisitions (non-ionic iodinated contrast agent, 100 mL at 3 mL/s for portal venous phase imaging), with 5 mm slice thickness and standard body reconstruction kernels. Protocol selection (contrast vs. non-contrast) was determined by the requesting clinician based on clinical indication and patient history; this heterogeneity in acquisition is acknowledged as a limitation that may affect detection rates for certain finding types (e.g., small adrenal lesions, renal masses).

### 2.2. Study Population

**Figure 1:**
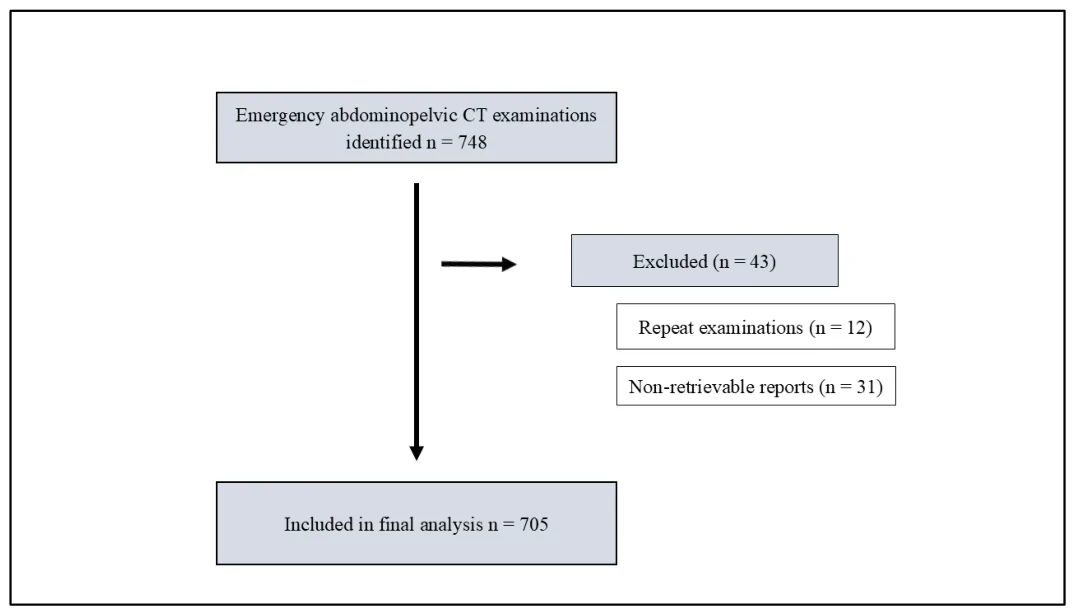
Flow diagram illustrating the selection of the study population.

All consecutive emergency abdominopelvic CT examinations performed between 1 January 2023 and 31 December 2025 were eligible for inclusion. No age restriction was applied; both adult and pediatric examinations were considered. A total of 748 emergency abdominopelvic CT examinations were initially identified. Of these, 43 were excluded, comprising 12 repeat examinations on the same patient during the study period (only the first examination was retained) and 31 examinations with non-retrievable consultant-approved reports. The remaining 705 unique examinations met the inclusion criteria and were reviewed (Figure 1).

### 2.3. Identification of Incidental Findings

Incidental findings were defined a priori as imaging findings unrelated to the clinical indication for which the CT examination was requested. Only findings documented in the consultant-approved final radiology report were considered, in keeping with prior pragmatic studies of report-documented incidental finding rates [2,7]. The determination of whether a finding was incidental was based on the consultant radiologist’s interpretation as documented in the final report; the study team did not independently determine whether findings were related to the presenting complaint or reinterpret the CT examinations. Findings related to the presenting complaint or to the acute indication were excluded.

### 2.4. Image and Report Review

Two senior radiology residents (one R3 and one R4) independently extracted incidental findings from the consultant-approved radiology reports using the predefined terminology-standardization framework. Discrepancies in data extraction and classification were resolved by consensus, with unresolved cases adjudicated by a board-certified consultant radiologist. All 705 reports were screened in full, so that every examination contributed to the denominator; however, patient-level demographic and anthropometric variables (age, sex, nationality, and body mass index) were extracted only for examinations in which at least one incidental finding was documented. Demographic data are therefore available for the 138 examinations with incidental findings and not for the cohort as a whole, and all demographic analyses are restricted to that subgroup.

To assess agreement in data extraction and classification, a random sample of 14 consultant-approved reports was independently coded by both reviewers. Inter-rater agreement was evaluated using Cohen’s kappa (κ) coefficient. Independent coding of a random sample of 14 CT reports demonstrated strong agreement between the two reviewers (κ = 0.84). Because agreement was assessed on only 14 reports, this estimate carries wide uncertainty and should be interpreted as supportive rather than definitive evidence of coding reliability.

### 2.5. Terminology Standardization Framework

To reduce semantic variability arising from heterogeneous reporting practices and synonymous radiologic descriptors, IFs were harmonized using a predefined terminology-standardization framework while preserving anatomically meaningful distinctions. Anatomic variants were separated from pathological findings. The framework was applied uniformly across abdominal, genitourinary, gynecological, musculoskeletal, spinal, thoracic, and vascular systems. Key consolidations included the following:

- Midgut malrotation was classified as an incidental congenital gastrointestinal finding.
- Segment-specific bowel mural thickening (e.g., transverse colon, distal jejunum) was unified as bowel mural thickening.
- Dilated common bile duct and distended gallbladder were combined as biliary tract dilatation.
- Single and multiple gallbladder calculi were merged as gallbladder stones; analogous consolidation was applied to hepatic cysts, hepatic lesions, and renal cysts.
- Inguinal, umbilical, and hiatal variants were grouped as abdominal hernias.
- Renal, ureteric, and bladder calculi were grouped as urinary tract stones.
- Pelvicalyceal dilatation, hydronephrosis, and hydroureter were combined as upper urinary tract dilatation for summary analysis but reported separately in the detailed table.
- Adnexal complex cystic lesions were unified with adnexal cysts; nabothian and paravaginal cysts were grouped as pelvic cystic lesions.
- Disc bulges, lumbar/thoracic spondylosis, and spondylodegenerative disc disease were unified as degenerative spine/disc changes.
- Celiac, superior mesenteric, and renal arterial stenoses or occlusions were combined as arterial stenosis/occlusion.
- Less common or isolated entities, including pancreatic lipoma and other minor lesions, were placed in other categories to preserve dataset clarity.

Findings without laterality information or those not applicable to side-based classification were assigned to a Not specified category to maintain dataset completeness and methodological transparency.

### 2.6. Definition of Clinical Actionability

Clinical actionability was defined a priori as the presence of an explicit recommendation for further diagnostic evaluation or follow-up in the consultant-approved report. We deliberately avoided the term clinical significance because the determination of true clinical importance requires longitudinal outcome data that were not available in this retrospective design. The proportion of patients with at least one actionable IF therefore reflects routine reporting behavior rather than verified pathological importance.

### 2.7. Statistical Analysis

Data were analyzed using R version 4.3 (R Foundation for Statistical Computing, Vienna, Austria). Continuous variables are reported as mean ± standard deviation and as median with interquartile range (IQR), as appropriate. Categorical variables are reported as frequencies and percentages. Wilson 95% confidence intervals were calculated for headline proportions. The examination was the primary unit of analysis. The report-documented rate of incidental findings was calculated as the proportion of emergency abdominopelvic CT examinations containing at least one incidental finding (examination-level analysis). Clinical actionability was assessed at the patient (examination) level and was defined according to whether at least one incidental finding in an examination required additional evaluation or follow-up. The distribution of anatomical categories was calculated using the total number of individual incidental findings as the denominator (finding-level analysis). No inferential hypothesis testing was performed, consistent with the descriptive aims of the study.

### 2.8. Ethical Considerations

The study was approved by the Institutional Review Board of Jazan Health Cluster (Reference No. 2456, dated 28 August 2024). A waiver of informed consent was granted because of the retrospective design and the use of fully anonymized data. The study adhered to the principles of the Declaration of Helsinki.

### 2.9. Use of Generative Artificial Intelligence

Generative AI was used solely for language editing. Study design, data, analysis, and interpretation were entirely conducted by the authors.

## 3. Results

### 3.1. Cohort Characteristics

Of the 705 consecutive emergency abdominopelvic CT examinations reviewed during the 36-month study period, 138 (19.6%; 95% CI 16.8% to 22.7%) demonstrated at least one incidental finding. A total of 255 IFs were recorded among these examinations, with a mean of 1.85 findings per affected patient (range 1 to 5). The mean age of patients with IFs was 42.3 ± 18.2 years (median 40.5, IQR 30.3 to 56.0, range 3 to 85). Twelve patients (8.7%) were younger than 18 years. The mean body mass index was 26.6 ± 4.8 kg/m^2^ among the 126 adult patients and 26.0 ± 5.1 kg/m^2^ across all 138 patients with incidental findings. Seventy-eight patients (56.5%) were male, and 116 (84.1%) were Saudi nationals. Further work-up was explicitly recommended in 48 of 138 patients with IFs (34.8%; 95% CI 27.3% to 43.0%). Demographic characteristics are summarized in Table 1.

Pediatric examinations. Twelve of the 138 patients with incidental findings (8.7%) were younger than 18 years, with a median age of 13.5 years (range 3 to 17); nine were male and eleven were Saudi nationals. The mean body mass index in this subgroup was 19.8 ± 4.7 kg/m^2^. A documented recommendation for further evaluation was present in 4 of these 12 patients (33.3%; 95% CI 13.8% to 60.9%), similar to the 34.9% recorded among adult patients. Because incidental findings were recorded at the examination level without linkage to individual patients, the distribution of specific finding types within this subgroup could not be tabulated separately.

### 3.2. Distribution of Findings Across Major Anatomical Regions

Among the 255 incidental findings, 159 (62.4%) were located within the abdominopelvic cavity, 64 (25.1%) within the miscellaneous category (which encompasses spine, musculoskeletal, Immunity (lymphatic), and soft-tissue findings), and 32 (12.5%) within the thoracic and vascular region (Table 2). This distribution is consistent with the imaged field of view of standard emergency abdominopelvic CT protocols.

### 3.3. Distribution Across Secondary Anatomical Categories

Across secondary categories, the spine was the single most frequent site (38 findings; 14.9%), followed closely by the hepatobiliary system (37; 14.5%), genitourinary tract (31; 12.2%), and gynecological organs (26; 10.2%) (Table 3). Vascular findings, abdominal hernias, and gastrointestinal findings each accounted for 8% to 9% of the total. The least frequent categories were solid organs, soft tissues, and pancreatic findings, each representing fewer than 1.5% of total IFs.

### 3.4. Detailed Findings by Anatomical Category and Laterality

Table 4 presents the full inventory of incidental findings stratified by major region, secondary category, specific finding, and laterality. Within the abdominopelvic cavity, the most frequent specific findings were diffuse hepatic steatosis (15; 5.9%), inguinal hernia (11; 4.3%), and adnexal cysts (12; 4.7%). Within the miscellaneous category, degenerative spine and disc changes were dominant (13; 5.1%), followed by pelvic osseous lesions (11; 4.3%) and spondylolisthesis or foraminal compromise (9; 3.5%). Among thoracic and vascular findings, vascular anatomical variants (12; 4.7%) and arterial atherosclerotic or stenotic changes (8; 3.1%) were most common. Lateralized distribution was most evident for renal cysts, adnexal cysts, and pars defects, the latter showing predominant bilaterality (6 of 8 cases).

### 3.5. Most Frequent Incidental Findings

**Figure 2:**
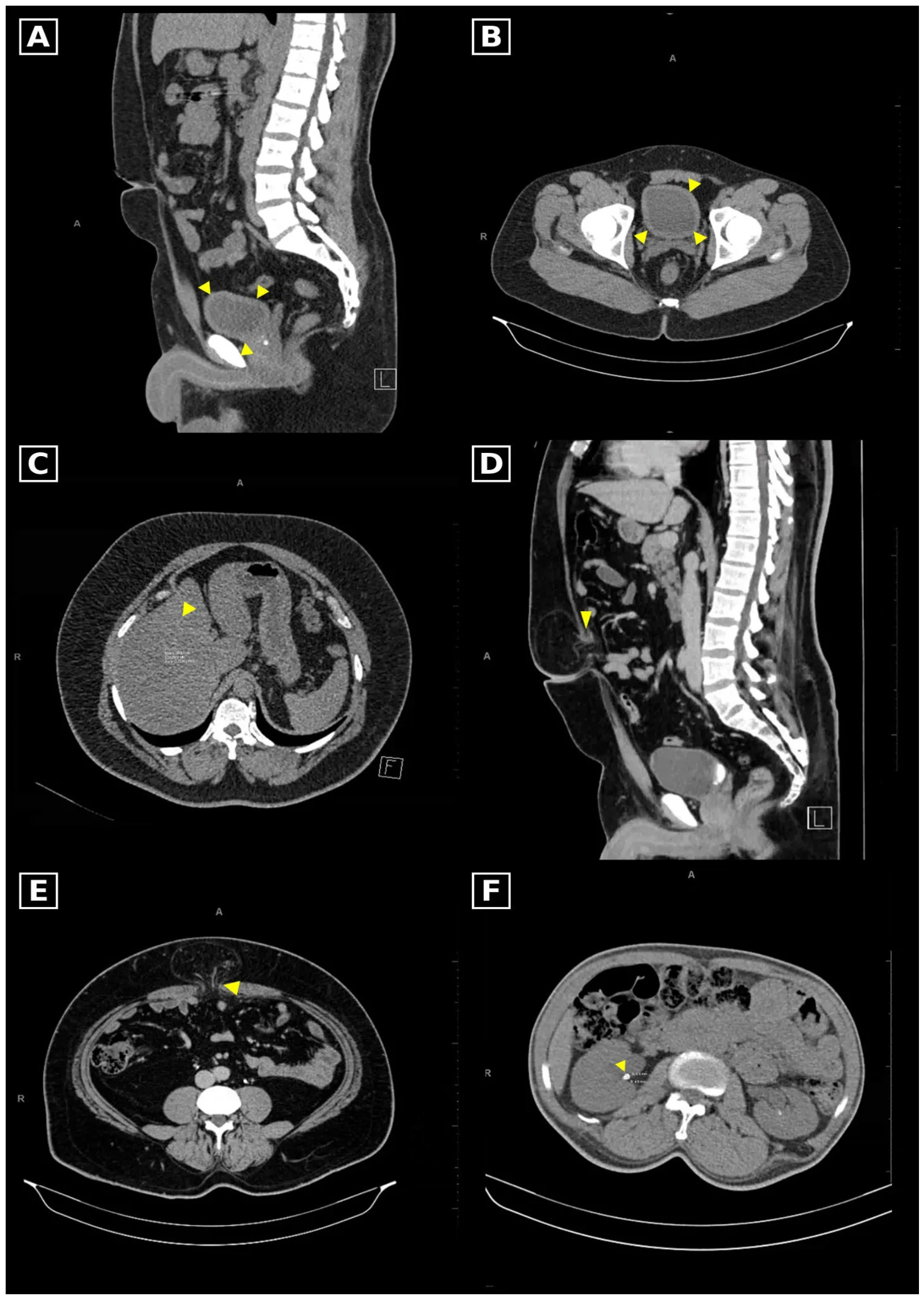
Representative CT images of selected incidental findings identified in the study. (**A**,**B**) Mild diffuse circumferential urinary bladder wall thickening in a 29-year-old man on sagittal and axial non-contrast CT images, respectively. (**C**) Diffusely decreased hepatic attenuation consistent with hepatic steatosis in a 42-year-old man on axial non-contrast CT. (**D**,**E**) Supraumbilical fat-containing hernia in a 43-year-old man on sagittal and axial contrast-enhanced CT images, respectively. (**F**) Right renal pelvic calculus measuring approximately 6 × 5 × 8 mm with an attenuation of 1340 HU in a 51-year-old man on axial non-contrast CT. Arrowheads indicate the relevant incidental findings.

To summarize the high-yield findings from the detailed inventory in Table 4, the eighteen most frequent incidental findings (each occurring five or more times) were tabulated separately (Table 5). Together, these accounted for 158 of 255 incidental findings (62.0%). Diffuse hepatic steatosis was the single most frequent specific finding (15; 5.9%), followed by degenerative spine and disc changes (13; 5.1%). Adnexal cysts and vascular anatomical variants were tied at 12 findings each (4.7%), and inguinal hernia and pelvic osseous lesions were tied at 11 each (4.3%). Spondylolisthesis or foraminal compromise occurred nine times (3.5%). Five findings occurred eight times each (3.1%): renal cysts, pars defect, bowel mural thickening, lymphadenopathy, and arterial atherosclerotic or stenotic changes. The remaining entries in Table 5 each occurred between five and seven times. Representative CT images of selected incidental findings are shown in Figure 2.

### 3.6. Recommendations for Further Evaluation

A documented recommendation for additional diagnostic evaluation or follow-up was present in 48 of 138 patients with IFs (34.8%; 95% CI 27.3% to 43.0%). Recommendations were most often associated with adrenal lesions, renal cysts that did not meet established benign criteria, indeterminate hepatic lesions, suspected vascular pathology, and incidentally detected lymphadenopathy. The remaining 90 patients (65.2%) were considered to have findings that did not require further evaluation in the context of routine clinical care.

### 3.7. Guideline-Anchored Management Recommendations

Each incidental finding was mapped to the management recommendation supported by current guidance and assigned to one of three action tiers (Table 6). Of 255 findings, 150 (58.8%) warranted further evaluation. Eleven (4.3%) met criteria for urgent or expedited assessment, comprising unexplained biliary dilatation, hydronephrosis and hydroureter, an indeterminate renal mass, arterial aneurysm above the surveillance threshold, and splenic infarction. A further 139 (54.5%) warranted routine or elective evaluation, most often metabolic and liver function assessment for hepatic steatosis, elective surgical referral for abdominal hernias, and size-stratified characterization of adrenal, adnexal and pelvic osseous lesions. The remaining 92 (36.1%) required no dedicated follow-up, dominated by degenerative spinal change and vascular anatomical variants. Recommendation varied by category: all adrenal, lymphatic and soft-tissue findings warranted some evaluation, whereas 27 of 38 spinal findings (71.1%) and 12 of 23 vascular findings (52.2%) required none. Genitourinary findings contributed 5 of the 11 urgent or expedited recommendations. Table 6 is constructed at finding level and is not directly comparable with the patient-level proportion reported in Section 3.6.

## 4. Discussion

### 4.1. Principal Findings

This retrospective study of 705 consecutive emergency abdominopelvic CT examinations from a tertiary referral hospital in southwestern Saudi Arabia identified incidental findings in 19.6% of scans, with a total of 255 distinct findings catalogued across 138 patients. Approximately one-third of affected patients had a documented recommendation for further evaluation. To our knowledge, this is among the first systematic descriptions of the spectrum of emergency CT incidental findings reported from the Jazan region.

### 4.2. Report-Documented Rates in Context

Reported rates of incidental findings on emergency abdominopelvic CT vary widely in the published literature, ranging from approximately 13% to over 60% [1,2,4,7,19,20]. This variability is reflected in a systematic review and meta-analysis by Evans et al., which included 69 studies comprising 147,763 emergency department encounters and reported a pooled incidental finding prevalence of 31.3%. The substantial between-study heterogeneity was attributed to differences in patient populations, imaging indications, study design, and the operational definitions of incidental findings [9]. Our patient-level report-documented rate of 19.6% is broadly consistent with the 20.5% reported by Lee et al. in young Korean adults [1] but is much lower than the 60.6% reported by Kelly et al. in an Irish ED cohort [2]. Several factors are likely responsible for this heterogeneity. First, definitions of incidentality differ across studies; some include any finding unrelated to the presenting complaint, whereas others restrict the count to findings of a predetermined importance [4,19,20]. Second, the unit of analysis varies: some studies report patient-level rates, whereas others report lesion counts, and the two are not directly comparable. Third, age is a strong determinant of incidental pathology; the median age of 57 years in the Kelly cohort is markedly higher than the median of 40 years in our cohort and would be expected to yield a higher rate of degenerative and vascular findings [2]. Finally, our reported rate reflects findings that were documented in consultant-approved reports rather than findings detectable on independent re-review. As Thompson et al. demonstrated, fewer than 10% of incidental findings are completely captured in routine ED documentation [8], suggesting that our figure is likely a lower bound on the true imaging prevalence.

### 4.3. Spectrum of Findings

The dominance of abdominopelvic-cavity findings (62.4%) is anatomically expected for an abdominopelvic CT protocol. At the secondary-category level, the prominence of spine (14.9%) and hepatobiliary (14.5%) findings reflect two converging phenomena: degenerative spinal pathology is captured incidentally in nearly any abdominopelvic acquisition, and hepatobiliary entities such as steatosis, simple hepatic cysts, and gallstones are highly prevalent in the general adult population. These observations align with Lee et al., who reported renal and hepatobiliary findings as the dominant entities in their young cohort [1], and with Kelly et al., in whose cohort gallstones, renal cysts, and hepatic cysts were the most frequent specific lesions [2]. The relatively lower contribution of thoracic and vascular findings in our study (12.5%) is consistent with the limited cephalad coverage of standard abdominopelvic CT protocols and contrasts with cohorts in which the imaging indication (e.g., MDCT urography for hematuria) prompts systematic inspection of extra-urinary structures [19].

Mapping the observed findings onto guideline criteria suggests that a larger share of findings would qualify for further evaluation than was documented in routine reports. Because the guideline mapping is performed at finding level and the documented rate is a patient-level proportion, the two figures cannot be subtracted, and we do not interpret the difference as a quantified shortfall in reporting. What the mapping does show is where guidance is most likely to be relevant in this setting: adrenal, lymphatic, genitourinary and hepatobiliary findings, which together account for a disproportionate share of tier-one and tier-two recommendations despite representing a minority of all findings. Structured report templates targeting these four categories would be a proportionate first step.

### 4.4. Clinical Actionability

Approximately 35% of patients with at least one incidental finding had a documented recommendation for further evaluation. This proportion is conceptually similar to the 49% of indeterminate or worrisome findings reported by Waqas et al. [7], and to the 12.7% of “important” findings reported by Samim et al. in an ED cohort imaged for suspected renal colic [4]. Direct numerical comparison is limited by differences in case mix and in the operationalization of importance. We chose actionability rather than significance to acknowledge that our metric captures reporting behavior rather than verified clinical impact. This distinction matters: explicit follow-up recommendations may be over-applied in low-risk contexts because of medico-legal concerns [21] and conversely may be omitted when the radiologist judges the finding to be benign. Both biases attenuate the relationship between recorded recommendations and true clinical importance.

### 4.5. Implications for Practice

Two implications follow from these data. First, incidental findings are sufficiently common in emergency abdominopelvic CT to warrant systematic attention, including standardized terminology, structured report fields, and explicit communication pathways for follow-up. The American College of Radiology white paper on incidental findings provides a framework that could be adapted locally [3]. More recently, the joint ACR–American College of Emergency Physicians (ACEP) White Paper has emphasized best practices for the communication and management of actionable incidental findings in emergency department imaging, reinforcing the importance of standardized communication pathways and appropriate follow-up processes [22]. Second, given the divergence among published estimates, comparisons across institutions are meaningful only when the unit of analysis, the definition of incidentality, and the threshold for actionability are fully transparent. Future work should extend beyond detection to quantify downstream outcomes, including follow-up completion, diagnostic yield, costs, and patient-reported impact [23,24]. Linking structured reports to electronic-health-record alerts, as suggested by Brown [21], could help ensure that actionable findings are not lost between the ED encounter and outpatient follow-up.

A further limitation concerns the guideline mapping presented in Table 6. Several published algorithms are conditional on features not recorded in our dataset, including lesion diameter, unenhanced attenuation, Bosniak category and menopausal status. Findings were therefore assigned to the action tier appropriate to the most common presentation of each entity, which will misclassify individual cases in both directions. The mapping represents the recommendation that guidance would support a typical finding of that type, not an adjudicated recommendation for each specific patient, and it should not be read as a measure of reporting adequacy.

### 4.6. Strengths and Limitations

Strengths of this study include the consecutive sampling of emergency examinations over a 36-month period, the application of a predefined terminology-standardization framework, the explicit pre-specification of the unit of analysis, and the reporting of confidence intervals for headline prevalence. Several limitations should also be considered. First, the study was conducted at a single tertiary-care center, which limits generalizability to institutions with different ED case-mix, CT utilization thresholds, referral pathways, and reporting practices. Second, only findings documented in consultant-approved reports were included; the reported rate therefore represents real-world reporting rather than the true imaging prevalence detectable on independent re-review. Detection is also protocol dependent for some entities: diffuse hepatic steatosis, the most frequent single finding in this cohort, is most reliably assessed on unenhanced images using attenuation thresholds, and its identification on the portal venous phase of a mixed contrast and non-contrast protocol is insensitive and threshold dependent. The frequency of steatosis reported here is therefore likely to be an underestimate and is not standardized across protocols. In addition, detailed indication-level data for individual CT examinations were unavailable, precluding analyses stratified by imaging indication. Furthermore, because the study relied on report-level data rather than independent image review, granular application of ACR incidental finding criteria was not feasible. Accordingly, the assessment of clinical actionability was based on the information documented in the reports and may not fully reflect detailed guideline-based classification. Third, our definition of clinical actionability was tied to explicit recommendations and is methodologically transparent but may both under- and over-estimate true clinical importance, as discussed above. Fourth, no age restriction was applied, so the cohort included pediatric as well as adult examinations and may not be directly comparable to adult-only cohorts in the literature. The analysis dataset recorded age only for the 138 examinations with incidental findings, so an age-restricted report-documented rate was not calculated. Restricting the analysis of documented recommendations to the 126 adult patients gave 44 of 126 (34.9%; 95% CI 27.2% to 43.6%), materially identical to the whole-cohort value of 34.8%, indicating that inclusion of the pediatric examinations did not influence this conclusion. Fifth, the study was descriptive and not powered for inferential comparisons between subgroups. Finally, downstream outcomes such as diagnostic yield, treatment, and patient impact were beyond the scope of this analysis and remain priorities for future longitudinal work.

## 5. Conclusions

In this single-center retrospective study from southwestern Saudi Arabia, approximately one in five emergency abdominopelvic CT examinations contained at least one incidental finding, and one in three patients with such findings was referred for further evaluation. Spine, hepatobiliary, and genitourinary findings were the most frequently reported, consistent with the imaged field of view and with the underlying epidemiology of common adult pathology. The wide variability in published reported rate estimates appears to reflect methodological differences in patient selection, definitions of incidentality, and unit of analysis as much as true epidemiological variation. Future multicenter studies should evaluate standardized terminology, structured reporting approaches, and follow-up strategies to determine their impact on reporting consistency, downstream investigations, and patient outcomes.

## Figures and Tables

**Table 1:** Demographic and clinical characteristics of patients with incidental findings (n = 138).

**Variable**	**Mean ± SD or n (%)**
Age, years	42.3 ± 18.2
Median (IQR)	40.5 (30.3 to 56.0)
Range	3 to 85
BMI, kg/m^2^ (adults, n = 126)	26.6 ± 4.8
**Sex**	
Male	78 (56.5%)
Female	60 (43.5%)
**Nationality**	
Saudi	116 (84.1%)
Non-Saudi	22 (15.9%)
**Age group**	
Adult (≥18 years)	126 (91.3%)
Pediatric (<18 years)	12 (8.7%)
**Recommended further work-up**	
Yes	48 (34.8%)
No	90 (65.2%)

**Table 2:** Distribution of incidental findings across major anatomical regions (N = 255).

Major Anatomical Region	Count	Percentage
Abdominopelvic cavity	159	62.4%
Miscellaneous	64	25.1%
Thoracic and vascular	32	12.5%
**Total**	**255**	**100.0%**

**Table 3:** Distribution of incidental findings across secondary anatomical categories (N = 255).

Secondary Anatomical Category	Count	Percentage
Spine	38	14.9%
Hepatobiliary	37	14.5%
Genitourinary	31	12.2%
Gynecological	26	10.2%
Vascular	23	9.0%
Abdominal hernias	22	8.6%
Gastrointestinal	21	8.2%
Musculoskeletal	15	5.9%
Others	13	5.1%
Immunity (lymphatic)	8	3.1%
Adrenal	7	2.7%
Cardiopulmonary	7	2.7%
Solid organs	3	1.2%
Soft tissues	3	1.2%
Pancreatic	1	0.4%
**Total**	**255**	**100.0%**

**Table 4:** Incidental findings stratified by anatomical region, secondary category, specific finding, and laterality (N = 255). Percentages in the Total column are calculated using the total number of incidental findings as the denominator. Laterality percentages are calculated within each specific finding. (NS: not specified).

Major Region	Secondary Category	Finding	Total, n (%)	Side, n (%)			
				Right	Left	Bilateral	NS
Abdominopelvic cavity	Gastrointestinal	Bowel mural thickening	8 (3.1%)	0 (0.0%)	0 (0.0%)	0 (0.0%)	8 (100.0%)
		Diverticulosis	4 (1.6%)	0 (0.0%)	0 (0.0%)	0 (0.0%)	4 (100.0%)
		Redundant colon	3 (1.2%)	0 (0.0%)	0 (0.0%)	0 (0.0%)	3 (100.0%)
		Midgut malrotation	2 (0.8%)	0 (0.0%)	0 (0.0%)	0 (0.0%)	2 (100.0%)
		Subhepatic appendix	2 (0.8%)	0 (0.0%)	0 (0.0%)	0 (0.0%)	2 (100.0%)
		Appendicolith	2 (0.8%)	0 (0.0%)	0 (0.0%)	0 (0.0%)	2 (100.0%)
	Hepatobiliary	Diffuse hepatic steatosis	15 (5.9%)	0 (0.0%)	0 (0.0%)	0 (0.0%)	15 (100.0%)
		Hepatic lesions	6 (2.4%)	0 (0.0%)	0 (0.0%)	0 (0.0%)	6 (100.0%)
		Hepatomegaly	5 (2.0%)	0 (0.0%)	0 (0.0%)	0 (0.0%)	5 (100.0%)
		Gallbladder stones	4 (1.6%)	0 (0.0%)	0 (0.0%)	0 (0.0%)	4 (100.0%)
		Hepatic cysts	3 (1.2%)	0 (0.0%)	0 (0.0%)	0 (0.0%)	3 (100.0%)
		Biliary tract dilatation	2 (0.8%)	0 (0.0%)	0 (0.0%)	0 (0.0%)	2 (100.0%)
		Hepatic hemangioma	2 (0.8%)	0 (0.0%)	0 (0.0%)	0 (0.0%)	2 (100.0%)
	Pancreatic	Fatty pancreas	1 (0.4%)	0 (0.0%)	0 (0.0%)	0 (0.0%)	1 (100.0%)
	Abdominal hernias	Inguinal hernia	11 (4.3%)	2 (18.2%)	1 (9.1%)	4 (36.4%)	4 (36.4%)
		Umbilical hernia	6 (2.4%)	0 (0.0%)	0 (0.0%)	0 (0.0%)	6 (100.0%)
		Sliding hiatus hernia	4 (1.6%)	0 (0.0%)	0 (0.0%)	0 (0.0%)	4 (100.0%)
		Sliding inguinal hernia	1 (0.4%)	0 (0.0%)	1 (100.0%)	0 (0.0%)	0 (0.0%)
	Solid organs	Splenomegaly	2 (0.8%)	0 (0.0%)	0 (0.0%)	0 (0.0%)	2 (100.0%)
		Splenic infarction	1 (0.4%)	0 (0.0%)	0 (0.0%)	0 (0.0%)	1 (100.0%)
	Adrenal	Adrenal gland lesion	6 (2.4%)	4 (66.7%)	2 (33.3%)	0 (0.0%)	0 (0.0%)
		Adrenal gland hyperplasia	1 (0.4%)	0 (0.0%)	0 (0.0%)	1 (100.0%)	0 (0.0%)
	Genitourinary	Renal cysts	8 (3.1%)	2 (25.0%)	5 (62.5%)	1 (12.5%)	0 (0.0%)
		Genitourinary anatomical variants	7 (2.7%)	4 (57.1%)	2 (28.6%)	1 (14.3%)	0 (0.0%)
		Urinary tract stones	4 (1.6%)	0 (0.0%)	1 (25.0%)	1 (25.0%)	2 (50.0%)
		Pelvicalyceal dilatation	3 (1.2%)	3 (100.0%)	0 (0.0%)	0 (0.0%)	0 (0.0%)
		Hydronephrosis	3 (1.2%)	1 (33.3%)	2 (66.7%)	0 (0.0%)	0 (0.0%)
		Bladder wall thickening	2 (0.8%)	0 (0.0%)	0 (0.0%)	0 (0.0%)	2 (100.0%)
		Renal mass	1 (0.4%)	0 (0.0%)	1 (100.0%)	0 (0.0%)	0 (0.0%)
		Renal cortical scarring	1 (0.4%)	1 (100.0%)	0 (0.0%)	0 (0.0%)	0 (0.0%)
		Hydroureter	1 (0.4%)	1 (100.0%)	0 (0.0%)	0 (0.0%)	0 (0.0%)
		Bladder diverticulum	1 (0.4%)	0 (0.0%)	0 (0.0%)	0 (0.0%)	1 (100.0%)
	Gynecological	Adnexal cysts	12 (4.7%)	5 (41.7%)	7 (58.3%)	0 (0.0%)	0 (0.0%)
		Uterine fibroids	5 (2.0%)	0 (0.0%)	0 (0.0%)	0 (0.0%)	5 (100.0%)
		Endometrial thickening	3 (1.2%)	0 (0.0%)	0 (0.0%)	0 (0.0%)	3 (100.0%)
		Uterine morphological variation	3 (1.2%)	0 (0.0%)	0 (0.0%)	0 (0.0%)	3 (100.0%)
		Pelvic cystic lesions	2 (0.8%)	1 (50.0%)	0 (0.0%)	0 (0.0%)	1 (50.0%)
		Bulky uterus	1 (0.4%)	0 (0.0%)	0 (0.0%)	0 (0.0%)	1 (100.0%)
	Others	Other isolated findings	11 (4.3%)	2 (18.2%)	1 (9.1%)	0 (0.0%)	8 (72.7%)
Thoracic and vascular	Cardiopulmonary	Atelectatic changes	3 (1.2%)	2 (66.7%)	0 (0.0%)	1 (33.3%)	0 (0.0%)
		Cardiomegaly	1 (0.4%)	0 (0.0%)	0 (0.0%)	0 (0.0%)	1 (100.0%)
		Pericardial effusion	1 (0.4%)	0 (0.0%)	0 (0.0%)	0 (0.0%)	1 (100.0%)
		Pulmonary hypertension	1 (0.4%)	0 (0.0%)	0 (0.0%)	0 (0.0%)	1 (100.0%)
		Tracheal diverticulum	1 (0.4%)	0 (0.0%)	0 (0.0%)	0 (0.0%)	1 (100.0%)
	Vascular	Vascular anatomical variants	12 (4.7%)	1 (8.3%)	7 (58.3%)	1 (8.3%)	3 (25.0%)
		Arterial atherosclerotic/stenotic changes	8 (3.1%)	0 (0.0%)	1 (12.5%)	0 (0.0%)	7 (87.5%)
		Arterial aneurysm	3 (1.2%)	0 (0.0%)	0 (0.0%)	0 (0.0%)	3 (100.0%)
	Others	Other isolated findings	2 (0.8%)	1 (50.0%)	0 (0.0%)	0 (0.0%)	1 (50.0%)
Miscellaneous	Spine	Degenerative spine/disc changes	13 (5.1%)	0 (0.0%)	0 (0.0%)	0 (0.0%)	13 (100.0%)
		Spondylolisthesis/foraminal compromise	9 (3.5%)	0 (0.0%)	0 (0.0%)	0 (0.0%)	9 (100.0%)
		Pars defect	8 (3.1%)	1 (12.5%)	1 (12.5%)	6 (75.0%)	0 (0.0%)
		Congenital vertebral anomaly	3 (1.2%)	0 (0.0%)	0 (0.0%)	0 (0.0%)	3 (100.0%)
		Vertebral hemangioma	2 (0.8%)	0 (0.0%)	0 (0.0%)	0 (0.0%)	2 (100.0%)
		Vertebral osseous lesion	2 (0.8%)	0 (0.0%)	0 (0.0%)	0 (0.0%)	2 (100.0%)
		Vertebral endplate changes	1 (0.4%)	0 (0.0%)	0 (0.0%)	0 (0.0%)	1 (100.0%)
	Musculoskeletal	Pelvic osseous lesions	11 (4.3%)	2 (18.2%)	3 (27.3%)	3 (27.3%)	3 (27.3%)
		Abdominal wall laxity	2 (0.8%)	0 (0.0%)	0 (0.0%)	0 (0.0%)	2 (100.0%)
		Hip degenerative changes	2 (0.8%)	0 (0.0%)	2 (100.0%)	0 (0.0%)	0 (0.0%)
	Immunity (lymphatic)	Lymphadenopathy	8 (3.1%)	1 (12.5%)	0 (0.0%)	1 (12.5%)	6 (75.0%)
	Soft tissues	Subcutaneous nodule/mass	3 (1.2%)	1 (33.3%)	1 (33.3%)	0 (0.0%)	1 (33.3%)

**Table 5:** The eighteen most frequent incidental findings on emergency abdominopelvic CT (occurring ≥ 5 times), accounting for 158 of 255 incidental findings (62.0%).

Rank	Finding	Secondary Category	n (%)
1	Diffuse hepatic steatosis	Hepatobiliary	15 (5.9%)
2	Degenerative spine/disc changes	Spine	13 (5.1%)
3	Adnexal cysts	Gynecological	12 (4.7%)
4	Vascular anatomical variants	Vascular	12 (4.7%)
5	Inguinal hernia	Abdominal hernias	11 (4.3%)
6	Pelvic osseous lesions	Musculoskeletal	11 (4.3%)
7	Spondylolisthesis/foraminal compromise	Spine	9 (3.5%)
8	Renal cysts	Genitourinary	8 (3.1%)
9	Pars defect	Spine	8 (3.1%)
10	Bowel mural thickening	Gastrointestinal	8 (3.1%)
11	Lymphadenopathy	Immunity (lymphatic)	8 (3.1%)
12	Arterial atherosclerotic/stenotic changes	Vascular	8 (3.1%)
13	Genitourinary anatomical variants	Genitourinary	7 (2.7%)
14	Adrenal gland lesion	Adrenal	6 (2.4%)
15	Hepatic lesions	Hepatobiliary	6 (2.4%)
16	Umbilical hernia	Abdominal hernias	6 (2.4%)
17	Uterine fibroids	Gynecological	5 (2.0%)
18	Hepatomegaly	Hepatobiliary	5 (2.0%)

**Table 6:** Guideline-anchored management recommendations for incidental findings, by secondary anatomical category (N = 255).

Secondary Anatomical Category	IFs, n	Urgent or Expedited, n	Routine or Elective, n	No Dedicated Follow-Up, n	Principal Guideline-Anchored Recommendation	Guiding Reference
Spine	38	0	11	27	• Degenerative disc and endplate change, pars defect, congenital variant, typical vertebral hemangioma: clinical correlation only. • Spondylolisthesis or foraminal compromise: spine referral if symptomatic. • Indeterminate vertebral osseous lesion: MRI characterization.	[3]
Hepatobiliary	37	2	26	9	• Diffuse steatosis: metabolic and liver function assessment. • Indeterminate hepatic lesion: multiphasic CT or MRI, stratified by lesion size and malignancy risk. • Simple cyst, typical hemangioma: no follow-up. • Unexplained biliary dilatation: expedited MRCP with liver function testing.	[3,12,13]
Genitourinary	31	5	17	9	• Renal cyst: stratify by Bosniak category; no follow-up for categories I and II. • Hydronephrosis, hydroureter, indeterminate renal mass: expedited urological evaluation. • Urinary tract stone, bladder wall thickening: elective urology referral. • Anatomical variant, cortical scarring: no follow-up.	[3,14]
Gynecological	26	0	15	11	• Adnexal cyst: stratify by maximum diameter and menopausal status; ultrasound follow-up above the size threshold. • Endometrial thickening: gynecological referral, particularly after menopause. • Fibroid, uterine morphological variant, nabothian or paravaginal cyst: no follow-up.	[15]
Vascular	23	3	8	12	• Arterial aneurysm: triage by maximum diameter; vascular surgical referral above threshold, interval surveillance below. • Atherosclerotic or stenotic change: cardiovascular risk factor assessment, not imaging follow-up. • Anatomical variant: document for future surgical planning only.	[16]
Abdominal hernias	22	0	18	4	• Inguinal or umbilical hernia: elective general surgical referral; escalate only if obstruction or strangulation is suspected. • Sliding hiatus hernia: clinical correlation for reflux symptoms only.	[3]
Gastrointestinal	21	0	10	11	• Bowel mural thickening: clinical correlation; consider endoscopy or CT enterography. • Midgut malrotation: surgical referral given volvulus risk. • Diverticulosis, redundant colon, subhepatic appendix, appendicolith: no follow-up if asymptomatic.	[3]
Musculoskeletal	15	0	11	4	• Indeterminate pelvic osseous lesion: radiography or MRI; escalate if a known primary malignancy is present. • Hip degenerative change, abdominal wall laxity: no follow-up.	[3]
Others	13	–	–	–	• Heterogeneous isolated entities, managed individually. Not assigned to a tier.	–
Immunity (lymphatic)	8	0	8	0	• Lymphadenopathy: assess short-axis diameter, nodal architecture, enhancement, and distribution. • Nodes meeting abnormal criteria: clinical correlation and evaluation for an infective, inflammatory, or neoplastic cause.	[17]
Adrenal	7	0	7	0	• Lesion below 4 cm with unenhanced attenuation under 10 HU: no follow-up. • Indeterminate lesion: adrenal washout CT or chemical-shift MRI. • Suspected hormonal activity or hyperplasia: biochemical evaluation.	[18]
Cardiopulmonary	7	0	3	4	• Cardiomegaly, pericardial effusion, signs of pulmonary hypertension: echocardiography and cardiology referral. • Atelectatic change, tracheal diverticulum: no follow-up.	[3]
Solid organs	3	1	2	0	• Splenic infarction: expedited evaluation for an embolic, hematological, or vasculitic source. • Splenomegaly: hematological and hepatic assessment.	[17]
Soft tissues	3	0	3	0	• Indeterminate subcutaneous nodule or mass: ultrasound characterization; excision or biopsy if features are atypical.	[3]
Pancreatic	1	0	0	1	• Fatty pancreas: benign, no follow-up.	[3]
**Total**	**255 (100)**	**11 (4.3)**	**139 (54.5)**	**92 (36.1)**	**Overall, 150 of 255 incidental findings (58.8%) would warrant some form of further evaluation under current guideline criteria.**	

## Data Availability

The data presented in this study is available on request from the corresponding author. The data are not publicly available due to ethical restrictions and privacy concerns related to sensitive patient health information.

## Abbreviations

The following abbreviations are used in this manuscript:

ACR	American College of Radiology
BMI	Body Mass Index
CT	Computed Tomography
ED	Emergency Department
IF	Incidental Finding
MDCT	Multidetector Computed Tomography

## References

[1] Lee K., Jung K.U., Lee C., Lee D. (2025). Incidental Findings on Abdominopelvic CT in Young Korean Soldiers: Prevalence, Clinical Relevance, and Healthcare System Implications. Healthcare.

[2] Kelly M.E., Heeney A., Redmond C.E., Costelloe J., Nason G.J., Ryan J., Brophy D., Winter D.C. (2015). Incidental findings detected on emergency abdominal CT scans: A 1-year review. Abdom. Imaging.

[3] Berland L.L., Silverman S.G., Gore R.M., Mayo-Smith W.W., Megibow A.J., Yee J., Brink J.A., Baker M.E., Federle M.P., Foley W.D. (2010). Managing incidental findings on abdominal CT: White paper of the ACR incidental findings committee. J. Am. Coll. Radiol..

[4] Samim M., Goss S., Luty S., Weinreb J., Moore C. (2015). Incidental findings on CT for suspected renal colic in emergency department patients: Prevalence and types in 5,383 consecutive examinations. J. Am. Coll. Radiol..

[5] Ather H., Faizullah K., Achakzai I., Siwani R., Irani F. (2009). Alternate and incidental diagnoses on noncontrast-enhanced spiral computed tomography for acute flank pain. Urol. J..

[6] Moore C.L., Daniels B., Singh D., Luty S., Molinaro A. (2013). Prevalence and clinical importance of alternative causes of symptoms using a renal colic computed tomography protocol in patients with flank or back pain and absence of pyuria. Acad. Emerg. Med..

[7] Waqas S., Johnson J.O., Salastekar N., Maddu K.K., Khosa F. (2014). Incidental findings detected on abdomino-pelvic multidetector computed tomography performed in the acute setting. Am. J. Emerg. Med..

[8] Thompson R.J., Wojcik S.M., Grant W.D., Ko P.Y. (2011). Incidental findings on CT scans in the emergency department. Emerg. Med. Int..

[9] Evans C.S., Arthur R., Kane M., Omofoye F., Chung A.E., Moreton E., Moore C. (2022). Incidental radiology findings on computed tomography studies in emergency department patients: A systematic review and meta-analysis. Ann. Emerg. Med..

[10] Sabiq S., Alzauir A., Alenizi S.A. (2024). Incidental computed tomography findings among traumatized adults: A one-year analysis at a trauma center. Cureus.

[11] von Elm E., Altman D.G., Egger M., Pocock S.J., Gøtzsche P.C., Vandenbroucke J.P., STROBE Initiative (2007). The Strengthening the Reporting of Observational Studies in Epidemiology (STROBE) statement: Guidelines for reporting observational studies. Lancet.

[12] Gore R.M., Pickhardt P.J., Mortele K.J., Fishman E.K., Horowitz J.M., Fimmel C.J., Talamonti M.S., Berland L.L., Pandharipande P.V. (2017). Management of incidental liver lesions on CT: A white paper of the ACR Incidental Findings Committee. J. Am. Coll. Radiol..

[13] Sebastian S., Araujo C., Neitlich J.D., Berland L.L. (2013). Managing incidental findings on abdominal and pelvic CT and MRI, part 4: White paper of the ACR Incidental Findings Committee II on gallbladder and biliary findings. J. Am. Coll. Radiol..

[14] Herts B.R., Silverman S.G., Hindman N.M., Uzzo R.G., Hartman R.P., Israel G.M., Baumgarten D.A., Berland L.L., Pandharipande P.V. (2018). Management of the incidental renal mass on CT: A white paper of the ACR Incidental Findings Committee. J. Am. Coll. Radiol..

[15] Patel M.D., Ascher S.M., Paspulati R.M., Shanbhogue A.K., Siegelman E.S., Stein M.W., Berland L.L. (2013). Managing incidental findings on abdominal and pelvic CT and MRI, part 1: White paper of the ACR Incidental Findings Committee II on adnexal findings. J. Am. Coll. Radiol..

[16] Khosa F., Krinsky G., Macari M., Yucel E.K., Berland L.L. (2013). Managing incidental findings on abdominal and pelvic CT and MRI, part 2: White paper of the ACR Incidental Findings Committee II on vascular findings. J. Am. Coll. Radiol..

[17] Heller M.T., Harisinghani M., Neitlich J.D., Yeghiayan P., Berland L.L. (2013). Managing incidental findings on abdominal and pelvic CT and MRI, part 3: White paper of the ACR Incidental Findings Committee II on splenic and nodal findings. J. Am. Coll. Radiol..

[18] Mayo-Smith W.W., Song J.H., Boland G.L., Francis I.R., Israel G.M., Mazzaglia P.J., Berland L.L., Pandharipande P.V. (2017). Management of incidental adrenal masses: A white paper of the ACR Incidental Findings Committee. J. Am. Coll. Radiol..

[19] Song J.H., Beland M.D., Mayo-Smith W.W. (2012). Incidental clinically important extraurinary findings at MDCT urography for hematuria evaluation: Prevalence in 1209 consecutive examinations. Am. J. Roentgenol..

[20] Morgan A.E., Berland L.L., Ananyev S.S., Lockhart M.E., Kolettis P.N. (2015). Extraurinary incidental findings on CT for hematuria: The radiologist’s role and downstream cost analysis. Am. J. Roentgenol..

[21] Brown S.D. (2013). Professional norms regarding how radiologists handle incidental findings. J. Am. Coll. Radiol..

[22] Moore C.L., Baskin A., Chang A.M., Cheung D., Davis M.A., Fertel B.S., Hans K., Kang S.K., Larson D.M., Lee R.K. (2023). White paper: Best practices in the communication and management of actionable incidental findings in emergency department imaging. J. Am. Coll. Radiol..

[23] O’Sullivan J.W., Muntinga T., Grigg S., Ioannidis J.P.A. (2018). Prevalence and outcomes of incidental imaging findings: Umbrella review. BMJ.

[24] Ding A., Eisenberg J.D., Pandharipande P.V. (2011). The economic burden of incidentally detected findings. Radiol. Clin. N. Am..

